# *Candida* and Host Determinants of Susceptibility to Invasive Candidiasis

**DOI:** 10.1371/journal.ppat.1003079

**Published:** 2013-01-03

**Authors:** Michail S. Lionakis, Mihai G. Netea

**Affiliations:** 1 Fungal Pathogenesis Unit, Laboratory of Clinical Infectious Diseases, National Institute of Allergy and Infectious Diseases, National Institutes of Health, Bethesda, Maryland, United States of America; 2 Department of Medicine, Nijmegen Institute for Infection, Inflammation and Immunity (N4i), Radboud University Nijmegen Medical Center, Nijmegen, The Netherlands; Duke University Medical Center, United States of America

## Introduction

*Candida* is the most common human fungal pathogen and the cause of invasive candidiasis, the fourth leading cause of nosocomial bloodstream infection in the United States with an estimated annual cost of ∼US$2 billion and mortality that exceeds 40% despite administration of antifungal therapy in modern intensive care unit facilities [1]. Hence, invasive candidiasis is an unmet medical condition for which better understanding of its pathogenesis at the host–pathogen interface is essential to improve patient outcomes. To that end, a mouse model of the infection, which introduces *Candida* yeast cells intravenously and mimics skin-derived bloodstream human candidiasis, has been successfully employed to study fungal and host factors that regulate susceptibility to the infection [2].

## Which *Candida* Virulence Factors Influence the Outcome of Invasive Candidiasis?

*Candida* expresses a variety of virulence factors that contribute to its pathogenesis and could be exploited for development of vaccines and targeted therapeutic strategies. Firstly, *Candida albicans* filaments' virulence factors, including secreted aspartyl proteases and phospholipases, are thought to be important for *Candida* invasion in infected organs and, probably, for mediating fungus-induced tissue immunopathology [3]. Secondly, *Candida* is able to efficiently adhere to and invade epithelial and endothelial cells via induced endocytosis and active penetration; both adhesion and invasion facilitate *Candida* dissemination [4]. Effective adhesion also enables *Candida* to form biofilms on implanted medical devices such as central venous catheters, which are a frequent portal of entry for invasive infection in humans [5]. Among the known *Candida* factors that promote its adhesion and invasion, the agglutinin-like sequence (Als) family has attracted significant attention; Als3 in particular, a *C. albicans*–specific virulence factor, was recently shown to mediate brain-specific *Candida* endothelial invasion and tissue penetration [6]. Specifically, increased surface expression of Als3 in the *vps51Δ/Δ C. albicans* mutant was shown to be responsible for its increased ability to invade brain endothelial cells in vitro and traffic to the brain in vivo via binding to the gp96 heat shock protein, which is expressed specifically on brain endothelium [6]. Als3 has formed the basis for the development of a cell wall protein-based vaccine strategy against candidiasis, which was recently tested safely in humans in a Phase I clinical trial [7]. Studies in mice revealed that IFN-γ and IL-17α produced by Th1 and Th17 lymphocytes were essential for vaccine-induced protection, via Ccl3- and Cxcl1-mediated neutrophil recruitment to sites of infection, which resulted in decreased *Candida* tissue burden [8].

Moreover, a fundamental *C. albicans* virulence factor is its ability to transition between unicellular yeast cells and filamentous growth during infection; in fact, it is the interchange between these morphotypes that is critical for pathogenesis, as strains locked either in the yeast or the filamentous forms have attenuated virulence in vivo [9], [10]. Of note, this morphogenic transition is not a prerequisite for virulence in non-*albicans Candida* species such as *Candida glabrata*, which is an important cause of invasive candidiasis in humans even though it does not form hyphae. Strikingly, the ability of *C. albicans* to form filaments in vivo is tissue-specific [2]. Hence, organs that successfully inhibit *Candida* growth in mice such as the liver and spleen prevent *Candida* filamentation, whereas hyphal formation is abundant in the kidney, the target organ of murine disseminated candidiasis, where *Candida* proliferation is inexorable [2]. Therefore, identifying tissue-specific immunological factors and environmental cues that restrict *Candida* filamentation could lead to the discovery of novel therapeutic interventions.

## How Does the Innate Immune System Recognize *Candida*?

**Figure 1:**
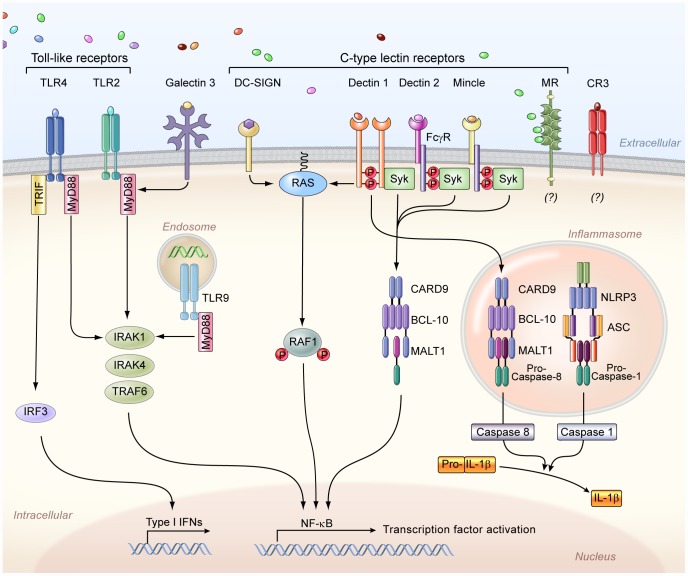
The principal cell surface pattern recognition receptors involved in *Candida* recognition. The Toll-like receptor 2 (TLR2) and TLR4 recognize phospholipomannans and *O*-linked mannans, respectively, whereas TLR9 within the cytosol recognizes fungal DNA, and intracellular TLR7 (not depicted) recognizes fungal RNA. TLR2 forms heterodimers with TLR1 or TLR6 for downstream signaling (not depicted), whereas TLR4 forms homodimers. Galectin-3, together with TLR2, recognizes β-mannosides. The membrane-bound C-type lectins dendritic cell–specific ICAM3-grabbing non-integrin (DC-SIGN), macrophage-inducible C-type lectin (Mincle), and macrophage mannose receptor (MR) recognize mannose-rich *Candida* structures. In addition, dectin-1 recognizes β-glucans, and dectin 2, together with the Fcγ receptor (FcγR), recognizes mannans. The complement receptor 3 (CR3) on neutrophils also recognizes β-glucans. Moreover, the NOD-like receptor NLRP3 (nucleotide-binding domain, leucine-rich-repeat-containing family, pyrin domain-containing 3) forms an inflammasome complex with ASC (apoptosis-associated speck-like protein containing a caspase recruitment domain) and caspase 1, which leads to interleukin-1β (IL-1β) production. In addition, downstream dectin-1 signaling through caspase recruitment domain-containing protein 9 (CARD9) leads to non-canonical inflammasome activation and IL-1β production via caspase 8. IFNs, interferons; NF-κB, nuclear factor-κB; Syk, spleen tyrosine kinase; IRF3, interferon regulatory factor 3; TRIF, TIR-domain-containing adapter-inducing interferon-β; MyD88, myeloid differentiation primary response gene 88; BCL-10, B-cell lymphoma/leukemia 10; MALT1, mucosa-associated lymphoid tissue lymphoma translocation protein 1; TRAF6, TNF receptor–associated factor 6; IRAK, interleukin-1 receptor-associated kinase.

The first step in mounting an effective anti-*Candida* immune response is fungal recognition by the innate immune system. Over the past decade there has been an explosion in our understanding of how soluble and membrane-bound pattern recognition receptors (PRRs) recognize various pathogen-associated molecular patterns (PAMPs) of *Candida* yeast and filamentous forms (Text S1) (reviewed in [11], [12]). In brief, the complement components C3 and C5, the complement receptor 3 (CR3), the Toll-like receptors (TLR)-2 (in interaction with TLR1 and TLR6), TLR4, TLR7, and TLR9, and the C-type lectins (CLRs) dectin-1, dectin-2, mannose receptor, DC-SIGN, and Mincle are among the PRRs shown to recognize different fungal PAMPs including mannan, β-glucan, RNA, and DNA (Figure 1); several of these PRRs are indispensable for host defense in vivo by inducing the secretion of pro-inflammatory cytokines and chemokines and modulating innate and adaptive antifungal immune responses (Text S1) [11], [12]. In fact, synergistic interactions between different PRRs resulting in augmented downstream immune activation have been demonstrated, such as between TLRs and CLRs or C5a and TLRs [11], [12]. *Candida* also activates the inflammasome; both the Nlrp3/caspase-1 pathway and the non-canonical caspase-8 pathway have been implicated in IL-1β production via dectin-1/syk activation by β-glucans (Figure 1) [12], [13].

The challenge for future research will be to define how *Candida* recognition is integrated by the array of different PRRs in vivo and to determine what is the relative contribution of individual PRRs on different myeloid cells (e.g., dectin-1 or CR3 as β-glucan receptors on neutrophils versus monocytes/macrophages versus dendritic cells) in modulating downstream anti-*Candida* immune responses. In addition, more studies are needed to understand how *Candida* influences its recognition in vivo by employing immune evasion strategies; for example, β-glucan exposure on the *Candida* surface occurs in infected mouse tissues only late after infection [14], thus preventing CLR-mediated pathogen recognition during the early phase of invasive infection, when recruitment of effector immune cells is critical for survival [15].

In addition, the notable diversity in PAMP structure among various *Candida* experimental strains impedes on drawing definite conclusions about the in vivo role of certain PRRs, as apparently discrepant results have been reported for some TLRs and CLRs with different fungal strains [11], [12], [16]. To that end, the logistical and economical constraints associated with testing large numbers of *Candida* strains in mammals could potentially be ameliorated by employing non-vertebrate (e.g., *Drosophila melanogaster*) and/or mini-vertebrate model hosts (e.g., zebrafish) that have evolutionarily conserved innate immune pathways (e.g., TLR signaling), and could allow for facile and inexpensive high-throughput screening of greater numbers of *Candida* strains [17]. Not surprisingly, as *C. albicans* is the most common agent of human invasive candidiasis, research performed until now has predominantly focused on the recognition of this species, and much less is known about the interaction of other *Candida* species with the immune system. It is expected that research aiming to gain more insight on the recognition of emerging non-*albicans Candida* species will represent an important area of research in the coming years.

## What Are the Divergent Roles of Neutrophils during Invasive Candidiasis?

Neutrophils are indispensable for host defense against invasive candidiasis, and neutropenia is a well-recognized risk factor for development of and adverse outcome after infection in humans [1]. The protective effects of neutrophils are mediated via oxidative and non-oxidative mechanisms that result in efficient *Candida* killing [12]. Specifically, *Candida* ingestion is followed by assembly of the nicotinamide adenine dinucleotide phosphate (NADPH) oxidase complex at the phagosomal membrane and oxidative burst, which leads to generation of candidacidal reactive oxygen species and K^+^-influx–induced activation of neutrophil candidacidal granular proteases [12]. Neutrophils are the only effector cells able to inhibit *Candida* yeast-to-hyphae conversion, a process dependent on the oxidative burst [12]. The oxidative burst is also important for generation of neutrophil extracellular traps, which ensnare *Candida* yeasts and hyphae and appear important for anti-*Candida* host defense in vivo [18].

Nonetheless, neutrophils have differential effects in invasive candidiasis in vivo depending on the phase of the infection in mice. Specifically, early neutrophil presence after infection is protective, whereas neutrophil presence late after infection is pathogenic [15]. The requirement of early neutrophil accumulation for effective host defense appears to correlate with the organ-specific microbiological progression of invasive candidiasis [2]; thus, liver and spleen, which recruit large numbers of neutrophils early post-infection, effectively control fungal growth. Conversely, sluggish early accumulation of neutrophils in the kidney is associated with unabated fungal proliferation [2]. With regard to the pathogenic role of neutrophils late after infection, we recently reported that the chemokine receptor Ccr1 drives neutrophil-induced tissue immunopathology and mortality in invasive candidiasis by mediating neutrophil trafficking from the blood into the kidney during the late phase of the infection [19]. In addition, late neutrophil accumulation into the infected kidney was shown to depend on type I interferon signaling, which mediated tissue immunopathology and mortality [20]. Future research will be required to define the role of regulatory T cells and anti-inflammatory mediators including cytokines in restricting neutrophil-mediated tissue injury in invasive candidiasis. Of interest, in a subset of patients with invasive candidiasis, neutrophils exhibit pathogenic effects, as recovery from neutropenia is associated with worsening symptoms that requires corticosteroid administration [21]. Hence, identification of Ccr1, Ifnar1, and other molecular factors that mediate pathogenic neutrophil effects in invasive candidiasis could potentially lead to targeted therapeutic interventions in selected patients.

## What Is the Role of Other Immune Cell Types in Host Defense against Invasive Candidiasis?

Besides neutrophils, monocytes/macrophages are key phagocytic cells for protection against invasive candidiasis, as their depletion in mice leads to increased mortality [22]. Monocytes/macrophages are very effective in *Candida* phagocytosis and secretion of a variety of pro-inflammatory cytokines and chemokines that orchestrate the antifungal innate immune response [12]. Nevertheless, macrophages are significantly less able to inhibit yeast germination and kill *Candida* compared to neutrophils; the lack of macrophage myeloperoxidase and extracellular trap formation may, at least in part, account for this deficit [12]. On the other hand, the concomitant release of superoxide and nitric oxide, with the subsequent formation of peroxinitrite, has been suggested to mediate the macrophage anti-*Candida* effects in mice; yet, the importance of *Candida*-induced nitric oxide formation in human phagocytes is unclear [12]. Several studies have demonstrated the priming role of Th1 cytokines and the inhibitory role of Th2 cytokines on the macrophage killing capacity, but more research is required to elucidate the molecular mechanisms of macrophage activation and effector function in vivo at the sites of *Candida* infection. In addition, future studies should aim to shed light on the relative role of recruited versus resident monocytes/macrophages in host defense against invasive candidiasis.

Intriguingly, besides the protective role of monocytes/macrophages in host defense against primary *Candida* challenge, monocytes were recently shown to also confer protection following systemic *Candida* re-infection via functional reprogramming that involves the dectin-1/Raf-1 non-canonical signaling pathway and results in enhanced cytokine production [23]. This monocyte/macrophage reprogramming that results in innate immune memory (termed “trained immunity”) appears to involve epigenetic mechanisms mediated via stable changes in histone trimethylation at H3K4 [23]. Thus, induction of trained immunity shows promise for the potential design of novel vaccination strategies against candidiasis.

The role of other hematopoietic cells in host defense against invasive candidiasis merits further investigation. For example, dendritic cells are able to phagocytize *Candida* yeast and hyphal forms and respond differentially to the *Candida* morphotypes by priming the production of distinct cytokines [24]. In addition, CD1d^+^ dendritic cells were recently shown to activate invariant natural killer T cells to mediate innate immune responses against fungi (including *Candida*) via dectin-1- and MyD88-dependent mechanisms without apparent requirement for fungal lipid antigen presentation [25]. However, the in vivo role of different dendritic cell subsets remains unclear, partly owing to technical difficulties in achieving specific dendritic cell depletion. Moreover, consistent with the lack of enhanced susceptibility of patients with acquired immunodeficiency syndrome, severe combined immunodeficiency, X-linked agammaglobulinemia, and common variable immunodeficiency to invasive candidiasis, T and B lymphocytes are dispensable for host defense in the mouse model [26]. Nonetheless, T lymphocytes play a critical role in protection against mucosal candidiasis [26]. In addition, the recent demonstration of direct IgA-mediated anti-*Candida* effects after systemic infection [27] and of protective Th17-mediated responses by *Candida*-specific CD4^+^ T cells after systemic infection of mice vaccinated with the Als-derived peptide pALS [28] suggest that exploiting antibody-mediated and cell-mediated anti-*Candida* immunity could lead to the design of effective vaccination and immunomodulatory strategies against invasive and mucosal candidiasis in patients.

## What Host Factors Mediate Protection against Invasive Candidiasis in Humans?

In agreement with the requirement of innate immunity for effective host defense in the mouse model of invasive candidiasis, certain innate immune factors have been associated with protection against the infection in humans [26]. Consistent with the crucial role of the NADPH oxidase in phagocyte killing and the heightened susceptibility of NADPH oxidase-deficient mice to invasive candidiasis [8], [12], patients with chronic granulomatous disease are at increased risk for development of the infection [26]. In addition, in line with the enhanced susceptibility of myeloperoxidase-deficient mice to invasive candidiasis and the impaired anti-*Candida* killing capacity of human myeloperoxidase-deficient phagocytes, invasive candidiasis occurs in patients with myeloperoxidase deficiency, the most common inherited phagocytic disorder [26]. Yet, the majority of myeloperoxidase-deficient patients are asymptomatic, and invasive candidiasis develops only in patients with autosomal-recessive complete myeloperoxidase deficiency who also have concomitant disorders that adversely affect phagocyte function such as diabetes [26].

On the other hand, there is no universal concordance between humans and mice in regard to the importance of fungal PRRs and pro-inflammatory cytokines in host defense against invasive candidiasis. Hence, complement deficiencies and MyD88 mutations do not appear to confer a significant risk for invasive candidiasis in humans as opposed to mice [26]; however, a recent large cohort study demonstrated that TLR1 single nucleotide polymorphisms in Caucasian patients were associated with heightened risk for development of invasive candidiasis, suggesting that TLR signaling may contribute to optimal anti-*Candida* immunity in humans [29]. Furthermore, mutations in the adaptor molecule CARD9 predispose to both invasive and chronic mucocutaneous candidiasis (Text S1) [26], whereas dectin-1 mutations appear to be associated with chronic mucocutaneous but not invasive candidiasis in humans; instead, *Card9^−/−^* and *dectin-1^−/−^* mice are both susceptible to invasive candidiasis (Text S1) [26] Finally, Johnson and colleagues recently examined the impact of cytokine polymorphisms on host susceptibility to invasive candidiasis and reported that polymorphisms in IL-10 and IL-12B, but not IL-1β or IFN-γ, were associated with persistent fungemia in patients with candidemia [30]. Future studies will be required to identify additional genetic risk factors for development of and/or adverse outcome after invasive candidiasis, as such knowledge could eventually lead to individualized risk stratification and prognostication strategies for patients.

## Perspective

*Candida* is a commensal organism that colonizes 50% of individuals of a population at any given time, but in conditions leading to weakening of host defense mechanisms it can convert to an opportunistic pathogen causing localized mucosal disease or life-threatening invasive infections with high mortality rate despite antifungal therapy. In recent years we have witnessed a surge of studies of *Candida* pathogenesis at the host–pathogen interface. Dissecting the fungal virulence factors that foster the transition of *Candida* from a commensal to an opportunistic pathogen, and deepening our understanding of the molecular and cellular basis of effective antifungal immunity should lead to novel risk stratification, prognostication, vaccination, and therapeutic strategies in patients.

## Supporting Information

Text S1 Additional important references on *Candida* and host determinants of susceptibility to invasive candidiasis that could not be cited in the text due to space constraints.
(DOCX)
