# Supplementary material for: Candida and Host Determinants of Susceptibility to Invasive Candidiasis
Source: PLoS Pathog. 2013 Jan 3;9(1):e1003079. doi: 10.1371/journal.ppat.1003079 (PMC3536687; doi:10.1371/journal.ppat.1003079)
Supplement: Text S1 — Additional important references on Candida and host determinants of susceptibility to invasive candidiasis that could not be cited in the text due to space constraints. (DOCX) [file ppat.1003079.s001.docx]

1. Biondo C, Malara A, Costa A, Signorino G, Cardile F, et al (2012) Recognition of fungal RNA by TLR7 has a nonredundant role in host defense against experimental candidiasis. J Immunol in press.
2. Brothers KM, Newman ZR, Wheeler RT. (2011) Live imaging of disseminated candidiasis in zebrafish reveals role of phagocyte oxidase in limiting filamentous growth. Eukaryot Cell 10: 932-944.
3. Calderone RA, Fonzi WA. (2001) Virulence factors of *Candida albicans*. Trends Microbiol 9: 327-335.
4. Casadevall A, Pirofski LA. (2012) Immunoglobulins in defense, pathogenesis, and therapy of fungal diseases. Cell Host Microbe 11: 447-456.
5. Ferwerda B, Ferwerda G, Plantinga TS, Willment JA, van Spriel AB, et al. (2009) Human dectin-1 deficiency and mucocutaneous fungal infections. N Engl J Med 361: 1760-1767.
6. Gazendam RP, Drewniak A, Tool ATJ. Van Houdt M, Jansen MH, et al. (2012) Invasive fungal infection and impaired neutrophil killing in human CARD9 deficiency. J Clin Immunol 32(Suppl 1): S73.
7. Glocker EO, Hennigs A, Nabavi M, Schäffer AA, Woellner C, et al. (2009) A homozygous CARD9 mutation in a family with susceptibility to fungal infections. N Engl J Med 361: 1727-1735.
8. Gross O, Gewies A, Finger K, Schäfer M, Sparwasser T, et al (2006) Card9 controls a non-TLR signalling pathway for innate anti-fungal immunity. Nature 442: 651-656.
9. Gross O, Poeck H, Bscheider M, Dostert C, Hannesschläger N, et al. (2009) Syk kinase signalling couples to the Nlrp3 inflammasome for anti-fungal host defence. Nature 459: 433-436.
10. Kankkunen P, Teirilä L, Rintahaka J, Alenius H, Wolff H, et al. (2010) (1,3)-beta-glucans activate both dectin-1 and NLRP3 inflammasome in human macrophages. J Immunol 184: 6335-6342.
11. Lanternier F, Pathan S, Vincent Q, Liu L, Cypowij S, et al. (2012) Human invasive dermatophytic disease is caused by inborn errors of CARD9. J Clin Immunol; 32(Suppl 1): S72.
12. Lehrer RI, Cline MJ. (1969) Leukocyte myeloperoxidase deficiency and disseminated candidiasis: the role of myeloperoxidase in resistance to *Candida* infection. J Clin Invest 48: 1478-1488.
13. Mahanty S, Greenfield RA, Joyce WA, Kincade PW. (1988) Inoculation candidiasis in a murine model of severe combined immunodeficiency syndrome. Infect Immun 56: 3162-3166.
14. Reeves EP, Lu H, Jacobs HL, Messina CG, Bolsover S, et al. (2002) Killing activity of neutrophils is mediated through activation of proteases by K+ flux. Nature 416: 291-297.
15. Reimnitz DP, Herbst M, Sawalle-Belohradsky J, Groll A, Schlegel PG, et al. (2012) Persistent chronic Candida meningitis in a child with homozygous CARD9 gene (Q295X) mutation. J Clin Immunol 32(Suppl 1): S77.
16. Romani L. (2000) Innate and adaptive immunity in *Candida albicans* infections and saprophytism. J Leukoc Biol 68: 175-179.
17. Rosentul DC, Plantinga TS, Oosting M, Scott WK, Velez Edwards DR, et al. (2011) Genetic variation in the dectin-1/CARD9 recognition pathway and susceptibility to candidemia. J Infect Dis 204: 1138-1145.
18. Saijo S, Ikeda S, Yamabe K, Kakuta S, Ishigame H, et al. Dectin-2 recognition of alpha-mannans and induction of Th17 cell differentiation is essential for host defense against *Candida albicans*. (2010) Immunity 32: 681-691.
19. Spellberg B, Ibrahim AS, Edwards JE Jr, Filler SG. (2005) Mice with disseminated candidiasis die of progressive sepsis. J Infect Dis 192: 336-343.
20. Taylor PR, Tsoni SV, Willment JA, Dennehy KM, Rosas M, et al. (2007) Dectin-1 is required for beta-glucan recognition and control of fungal infection. Nat Immunol 8: 31-38.
21. Uzun O, Ascioglu S, Anaissie EJ, Rex JH. (2001) Risk factors and predictors of outcome in patients with cancer and breakthrough candidemia. Clin Infect Dis 32: 1713-1717.
22. van Bruggen R, Drewniak A, Jansen M, van Houdt M, Roos D, et al. (2009) Complement receptor 3, not Dectin-1, is the major receptor on human neutrophils for beta-glucan-bearing particles. Mol Immunol 47: 575-581.
23. Vázquez-Torres A, Balish E. (1997) Macrophages in resistance to candidiasis. Microbiol Mol Biol Rev 61: 170-192.
24. Vazquez-Torres A, Jones-Carson J, Balish E. (1996) Peroxynitrite contributes to the candidacidal activity of nitric oxide-producing macrophages. Infect Immun 64: 3127-3133.
25. von Bernuth H, Picard C, Jin Z, Pankla R, Xiao H, et al. (2008) Pyogenic bacterial infections in humans with MyD88 deficiency. Science 321: 691-696.
26. Winkelstein JA, Marino MC, Johnston RB Jr, Boyle J, Curnutte J, et al. (2000) Chronic granulomatous disease. Report on a national registry of 368 patients. Medicine (Baltimore) 79: 155-169.
